# A Case of Low-Grade Appendiceal Mucinous Neoplasm: The Role of Preoperative Imaging and Surgical Technique in Achieving Favorable Outcomes

**DOI:** 10.7759/cureus.65168

**Published:** 2024-07-23

**Authors:** Daniel A Meza-Martinez, Yeudiel Suro Santos, Samantha J Andrade-Ordoñez, Julio A Palomino-Payan, Brando J Fematt-Rodriguez

**Affiliations:** 1 General Surgery, Instituto Mexicano del Seguro Social, Hospital General de Zona No. 33, Monterrey, MEX

**Keywords:** low-grade dysplasia, conventional appendectomy, low grade appendiceal mucinous neoplasm, rare lesions in appendix, appendiceal mass

## Abstract

Appendiceal mucinous neoplasms may present without symptoms or with chronic pain in the right lower quadrant. This report describes a case of a 35-year-old woman who presented with chronic right lower quadrant pain and was found to have a low-grade appendiceal mucinous neoplasm (LAMN). Physical examination revealed localized tenderness in the right lower quadrant with no additional symptoms. Preoperative laboratory results were normal, and a CT scan revealed a cystic appendiceal lesion with an internal calcification, initially mistaken for a fecalith, which led to the decision for exploratory laparotomy. Intraoperative findings confirmed the presence of a cystic-like appendiceal lesion, and an open appendectomy was performed. Histopathological examination revealed a low-grade appendiceal mucinous neoplasm (LAMN) confined to the mucosa, without evidence of metastatic disease. The patient had an uneventful recovery and required no additional treatment. This case highlights that surgical intervention with proper technique for confined appendiceal neoplasms, combined with effective preoperative imaging and thorough histopathological examination, is crucial for diagnosis and effective management, ensuring favorable outcomes.

## Introduction

Neoplasms of the appendix are classified into a range of epithelial and nonepithelial tumors with varying malignant potentials. They are rare, with an age-adjusted annual incidence of about 6 cases per 1,000,000 individuals. The prognosis is variable, with long-term survival rates ranging from 10% to 90% [1]. A variety of histological types comprise appendiceal neoplasms, which are broadly categorized into the following categories: neuroendocrine neoplasms, which are non-epithelial; and epithelial neoplasms, which include mucinous neoplasms, goblet cell adenocarcinomas, colonic-type adenocarcinomas, and signet ring cell adenocarcinomas. These tumors are uncommon, making up less than 2% of surgically removed appendices and only 1% of all intestinal neoplasms. Some seemingly indolent tumors may exhibit aggressive characteristics [1,2]. Previous studies have used various terminologies for appendiceal mucinous neoplasms (AMN). Preceding classifications and guidelines have distinguished LAMNs from high-grade appendiceal neoplasms (HAMNs) as separate entities. In 2019, the WHO refined the classification and pTNM staging for appendiceal tumors [2].

Mucinous appendiceal tumors display a wide range of clinical behaviors, from nearly benign lesions to high-grade adenocarcinomas with poor five-year survival rates. LAMN patients may present with symptoms similar to appendicitis, including right iliac fossa pain, fever, nausea, and vomiting [3]. A key concern of this neoplasm is mucin seeding into the peritoneum, potentially causing pseudomyxoma peritonei (PMP), which is associated with high mortality rates [2,3]. Although there is no definitive consensus on the surgical approach for LAMN, a simple appendectomy is often deemed sufficient for tumors confined to the appendix [4]. Due to their unpredictable progression, LAMNs can present in various ways. In this article, we describe a case where chronic right lower quadrant pain exacerbated, mimicking acute appendicitis, which is an uncommon form of presentation.

## Case presentation

A 35-year-old female with no significant past medical history presented with the chief complaint of right iliac fossa pain that had been ongoing for four months. The pain was intermittent and colicky, occurring three to four times per week, and had significantly worsened in the three days prior to seeking medical attention. She did not report any associated gastrointestinal symptoms, such as nausea, vomiting, or changes in bowel habits, nor any systemic symptoms like fever or weight loss. Physical examination revealed localized tenderness in the right iliac fossa, without evidence of peritoneal irritation such as rebound tenderness or guarding.

Initial laboratory investigations, including a complete blood count and a metabolic panel, returned within normal limits, showing no signs of acute inflammation or metabolic abnormalities (Table 1).

**Table 1:** Laboratory results The patient's laboratory results were within the normal reference range.

Test	Result	Reference Values
Complete Blood Count		
Hemoglobin (g/dL)	13.5	12.0-15.5
Hematocrit (%)	42	36-46
White Blood Cells (x10^3/uL)	9.5	4.5-11
Neutrophils (%)	68	40-70
Eosinophils (%)	1	1-4
Monocytes (%)	3	2-8
Platelets (x10^3/uL)	251	150-450
Metabolic Panel		
Glucose (mg/dL)	90	70-100
Urea (mg/dL)	20	10-40
Creatinine (mg/dL)	0.7	0.6-1.1
Sodium (mEq/L)	139	135-145
Potassium (mEq/L)	4.1	3.5-5.0
Chloride (mEq/L)	102	98-107
Calcium (mg/dL)	9.1	8.5-10.5

**Figure 1:**
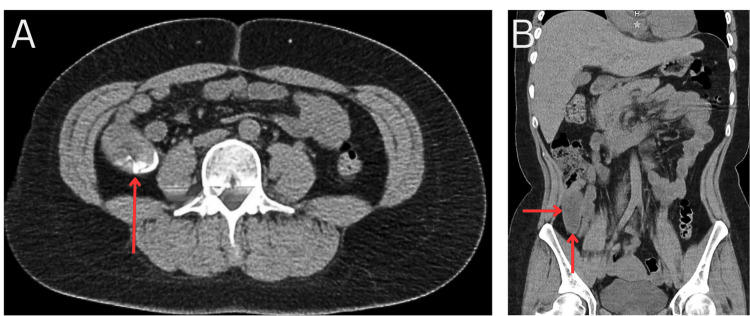
Preoperative CT scan demonstrating abnormal findings at the level of the cecal appendix A: Axial image showing an intraluminal calcification within the appendix, suggestive of an appendicolith (red arrow). B: Coronal image illustrating increased appendiceal diameter with homogeneous content, with no additional findings suggestive of acute appendicitis.

A preoperative abdominopelvic computed tomography (CT) scan was performed to evaluate the cause of her symptoms. The CT scan revealed a 28 x 21 mm calcified mass near the base of the cecal appendix coupled with appendiceal dilation, but no signs of acute inflammation or other conditions were observed, with the appendix measuring 74 x 27 x 26 mm (Figure 1). Taking these results and her clinical presentation into consideration, acute appendicitis was suspected, with the possibility of a fecalith as the origin of her condition.

Given this information, the decision to perform an exploratory laparotomy was made in order to address the suspected appendiceal pathology. Upon surgical examination, a cystic-like appendix measuring 8 x 3 x 3 cm was identified, with no evidence of inflammatory changes or other intra-abdominal abnormalities. Open appendectomy was performed through simple ligation of the appendix and stump using a 2-0 absorbable suture, and the rest of the procedure was completed without complications. The patient’s postoperative course was uneventful, and she was discharged 24 hours after surgery.

**Figure 2:**
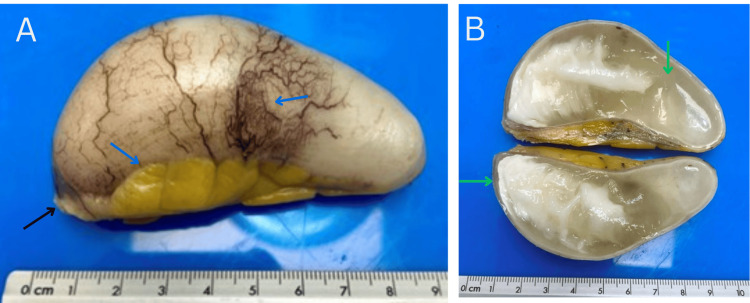
Macroscopic findings of the appendiceal specimen A: The cecal appendix measured 8 x 3.3 cm, and exhibited evident dilation. It had an intact external surface (black arrow), a whitish color, and a smooth appearance with congested vessels and minimal mesoappendiceal adipose tissue (blue arrows). B: On cross-sectional examination, the wall measured 2 mm and was macroscopically intact, with no evidence of diverticula or protrusions. The mucosal surface was smooth and pale white, and the cavity contained gelatinous material with some whitish regions (green arrows).

Upon receiving the pathology results, histopathological examination reported a cecal appendix that measured 8 x 3.3 cm, showing evident dilation with an intact external surface, a whitish and smooth appearance, congested vessels, and minimal mesoappendiceal adipose tissue (Figure 2). The section of the appendiceal wall measured 2 mm in thickness, with a macroscopically intact wall free of diverticula or protrusions. The mucosa was smooth and whitish, and the cavity was filled with gelatinous material interspersed with whitish areas. The resection margin was blunt and exhibited a soft consistency.

**Figure 3:**
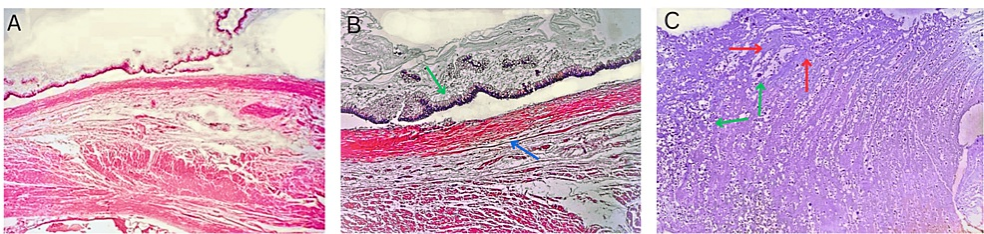
Histopathological examination showing findings consistent with LAMN A: Cross-section of the appendiceal wall. The mucosa and abundant mucin are seen, with an intact and thin lamina propria and a muscularis propria and adventitia with minimal mesoappendiceal tissue. No diverticula or infiltration into deeper layers of the appendix is noted (4x). B: Examination of the lumen, mucin, and epithelium. The epithelium is characterized by columnar cells with basal nuclei showing low-grade nuclear atypia, apical mucin-containing vacuoles (green arrow), and detachment of the epithelium. No infiltration into the lamina propria (blue arrow) or deeper appendiceal structures is observed (40x). C: Abundant mucin is observed in the appendiceal lumen (red arrows) along with some inflammatory cells (green arrows), but no mucinous epithelial cells are present (40x), confirming the diagnosis of LAMN. LAMN: low-grade appendiceal mucinous neoplasm

Microscopic analysis demonstrated abundant apical mucin with elongated nuclei and low-grade nuclear atypia, consistent with a low-grade mucinous neoplasm, which was confined to the mucosa, as there was no evidence of invasion into the lamina propria or deeper tissues, and no angiolymphatic or perineural invasion was observed (Figure 3).

After obtaining histopathologic results, the patient had a follow-up visit with oncology and general surgery, and given that her pathology was low-risk, additional follow-up was not deemed necessary.

## Discussion

LAMNs are rare, non-invasive epithelial tumors characterized by their non-infiltrative growth behavior and represent about 1% of all gastrointestinal tumors. Histologically, LAMNs are distinguished by a villous or flat proliferation of mucin-secreting epithelium with low-grade atypical features [2]. Despite their generally favorable outlook, the most significant complication associated with LAMNs is PMP, a severe condition resulting from mucin spreading into the peritoneal cavity or rupture during surgical procedures, which is linked to a high mortality risk [5]. Histopathological examination confirmed the diagnosis of a low-grade mucinous neoplasm confined to the mucosa. This finding is critical, as it delineates the neoplasm's behavior, indicating a lower risk of recurrence or dissemination compared to higher-grade lesions. The absence of invasion into the lamina propria, deeper layers, angiolymphatic or perineural spaces. Additionally, the pathology service remarkedly mentioned complete and negative resection margins with no mucin outside the appendiceal lumen, suggesting a favorable prognosis for this patient.

LAMNs are often discovered incidentally through radiological imaging or during surgery. Abdominal CT is often the most effective imaging examination for detecting appendiceal mucocele. It typically shows a distended appendix filled with fluid of low attenuation, with potential fine calcifications on the walls that become more visible after contrast enhancement. Although curvilinear mural calcification is highly indicative of mucocele, it is present in less than half of the cases. CT imaging also offers valuable insights into the anatomical relationships between the appendix and surrounding lower abdominal and pelvic structures, which is crucial for distinguishing mucocele from other conditions affecting the right ovary or fallopian tube, particularly when the mucocele extends into the pelvis. Additionally, CT is the preferred method for evaluating complications related to mucocele [6]. The preoperative CT findings in this patient were initially interpreted as a possible fecalith due to the presence of calcification and appendiceal dilation. However, the lack of acute inflammatory changes suggested an alternative diagnosis. This highlights the importance of considering neoplastic processes in patients with atypical appendiceal imaging findings, even in the absence of overt malignancy signs.

They may also present with vague symptoms, such as abdominal pain, palpable masses, or weight loss, and can mimic conditions like appendicitis, bowel obstruction, or a pelvic mass [7]. In this case, the patient presented with a four-month history of intermittent, colicky right iliac fossa pain, a symptom often associated with other common conditions such as irritable bowel syndrome or gynecological disorders. The absence of systemic symptoms and normal preoperative laboratory findings further complicated the diagnostic process in this case.

There is currently no consensus on definitive treatment guidelines for LAMNs, which fuels ongoing debates about the best surgical methods, adjuvant treatments, and follow-up care. The standard approach is typically to perform an appendectomy alone; however, in cases involving submucosal invasion, positive appendectomy margins, or lymph node involvement, a right hemicolectomy may be warranted [8]. The decision to perform an exploratory laparotomy was driven by the need for definitive diagnosis and treatment. Intraoperative findings of a cystic appendix without inflammatory changes pointed toward a mucinous neoplasm. The surgical approach involved an open appendectomy with meticulous dissection and ligation, ensuring complete resection, clear margins, and meticulous management of appendiceal mucin to prevent spillage and the risk of PMP.

The prognosis for LAMNs is influenced by the tumor stage, the extent of peritoneal mucin spillage, and the presence of PMP. Research indicates a notable prognostic distinction between cases with acellular versus cellular mucin, with recent studies suggesting that if perforation and extra-appendiceal mucin or cells are absent, the recurrence of the disease is highly uncommon. Nevertheless, patients with these pathological features must be carefully monitored [9]. In this case, the absence of risk factors for recurrence is demonstrated by the patient's continued well-being, with no evidence of disease, recurrence, or metastasis to date.

## Conclusions

This case demonstrates that a simple appendectomy can be an adequate treatment for LAMNs. It also reveals that clinical symptoms combined with imaging results may sometimes suggest an incorrect diagnosis. The careful management of the appendix and appendiceal stump during the appendectomy is key to successful outcomes in cases where there is no evidence of mucin intra-abdominally, as shown with this patient, demonstrated by no signs of recurrence or metastasis to date. This case underscores the importance of employing meticulous surgical techniques and conducting a detailed histopathological examination to ensure that no further treatment is needed and that the neoplasm is managed effectively.
